# The lure of the foreign stage: Chronicles of Italian mobility to France in the long eighteenth century

**DOI:** 10.12688/openreseurope.17027.1

**Published:** 2024-02-22

**Authors:** Elisa Cazzato

**Affiliations:** 1 Linguistics and Comparative Cultural Studies, Ca' Foscari University of Venice, Venice, Veneto, 30123, Italy

**Keywords:** artists migration, eighteenth century, theatre, performing arts, firework, circus

## Abstract

This article investigates the migration of three Italian-born artists working in France in the field of ephemeral entertainment and argues that their stories were part of a broader process of cultural history and artistic mobility of the long eighteenth century. These artists are the firework makers Ruggieri brothers, the circus performer Antonio Franconi and his family and the stage designer Ignazio Degotti. They left their home country ***at different points*** (1730s, 1750s and 1790 respectively), settling in Paris under different socio-political circumstances.

Due to the immaterial and contingent medium in which these artists chose to work, ***which is*** difficult to replicate and collect, the mobility of these artists has often remained a neglected story. To explore the reasons why these artists moved from their home country and the motivation that convinced them to stay in France, this research combines an attentive examination of archival material with a methodology influenced by methods of cultural history. The paper argues that their lives and their artistic expertise were not only aesthetically relevant, but also very much integrated within the defined social and cultural context they ***chose*** to live in.

## Introduction

For musicians and performers of the long eighteenth century, geographical mobility towards the major capitals of spectacle was a predictable move. Artists migrated for different reasons: forced by political or financial circumstances, intrigued by social prestige, or motivated by a network of established overseas connections. ^1^ For its lively and dynamic theatrical-life and for the diverse opportunities of popular entertainment, the city of Paris remained one of the most appealing capitals of spectacle, attracting the best artists and performers available on the market. ^2^ However, if migration of musicians, dramatists, and actors to France is often taken into scholarship account, ^3^ the mobility of those who worked as purveyors of ephemeral forms of entertainment like fireworks, circus performances, and stage sets, has remained a more neglected story. This is primarily due to the medium in which these artists chose to work, because unlike a music score or a written text, immaterial artistic expressions are difficult to replicate and collect. ^4^ Secondly, artists working backstage have been scholarly perceived as ‘subordinated’ to performers on stage, with the result that certain artistic careers have remained marginalized.

Through visual and written archival sources, this research focuses on the lives and careers of three Italian spectacle makers that migrated from Italy to France in different moments of the eighteenth century: these are the firework technicians Ruggieri brothers, the circus performer Antonio Franconi and his family, and the stage designer Ignazio Degotti. By highlighting their stories of mobility, this paper uncovers the reasons why they migrated and the motivations that made them stay. Moving from Daniel Roche’s scholarship on the “culture of mobility” in the modern era ^5^, this research is framed in a cross-cultural discourse that, to quote Mark Darlow's, is “concerned not just with the formal poetics and structure of the work of art but also its place within a specific field of signification and/or representation, and in a defined social and historical context.” ^6^

Methodologically speaking, this paper also conveys what was established by the historian Rahul Markovits in *Staging Civilization*. ^7^ In his book, the author unties the reasons why actors migrated away from their homeland, arguing that these reasons encompassed objective motivations (eg. conditions of employment and state of the market) as well as subjective personal reasons (eg. personal choices or connections). Markovits identifies “push” factors as those that led actors to depart, and “pull” factors as those that attracted them towards a specific destination. ^8^ He also distinguishes the “migration of discontinuity or rupture – in which immigrants invested emotionally in the space of the host country – from migration of continuity – in which the country of origin remained the frame of reference, and the space of the host country was only a means to an end.” ^9^

In this paper, after providing a concise overview of the state of theatrical affairs in Paris in the eighteenth century, I take Markovitz’s methodological frame as a reference to analyse the migration stories of the Ruggieris, Franconis, and Degotti from the Italian peninsula across the Alps. By exploring their careers as creators of spectacle, I examine the reasons behind their move and the motivations that encouraged them to stay. Finally, following Darlow’s statement, I argue how their lives and their artistic expertise were not only aesthetically relevant, but also very much integrated within the defined social and cultural context they choose to live in. Therefore, their stories should be considered as part of a broader process of cultural history and artistic mobility.

### Paris, a capital of spectacle

As examined by Jean-François Dubost in his attentive study *La France Italienne*, Italian immigration to France was very dynamic throughout the 16 ^th^ and 17 ^th^ centuries. ^10^ By examining this process in all its different forms and complexities, Dubost demonstrates how Italian immigrants were socially and financially well placed at all levels of French society. Their inventive and creative skills were acknowledged in a broad range of fields, tracing paths towards success. ^11^

Spectacle-makers that populated the French stage until the eighteenth century certainly fell into this category. For example, the celebrated Italian designers of the Baroque era Giacomo Torelli (1606–1678), Carlo Vigarani (1637–1713), and Niccolò Servandoni (1695–1766) were multifaceted artists whose careers took multiple trajectories. They were multi-talented artists, and their abilities as architects, stage designers, inventors of fireworks and machineries, and spectacle organizers were highly requested by the court society and their activities contributed in shaping the taste and expectations of French audiences. ^12^

Their position, however, was linked to aristocratic connections since they were hired by the Royal family as court artists and therefore the evolution of their careers depended on this specific environment. When the King had reasons to decide that it was time for a change, artists in his employ had to obey to his will, leave the country or eventually re-locate outside the court circles. The case of Torelli is a good example. His dismissal was decided by King Louis XIV after the designer accepted work for the courtesan Nicholas Fouquet, who had hired Torelli to set up a magnificent feast in his palace, which included a theatrical representation of the comédie-ballet *Les fâcheux* by Molière. The explicit purpose of the feast was to pay a lavish homage to the king, although - between the lines – Fouquet intended to exhibit the extent of his vast financial power. The result was that the king, intolerant of rivalry in luxe and opulence, did not appreciate Fouquet’s event. The courtesan was processed and anyone who had worked on the feast, like Torelli, was dismissed. Therefore, it can be argued that until the Revolution, court sociability and proper royal connections were the main path towards success in spectacle business and consequently, also the main reason to potentially be laid off.

In terms of theatre management until the Revolution, theatres were regulated by the Crown through a system of privileges and monopolies. These laws granted to the three *théâtres privilégiés* of Pairs, the Théâtre de l’Opéra (before the Revolution named Académie Royale de Musique), the Comédie-Italienne and the Comédie Française, the representation of specific repertoires that could not be staged elsewhere. ^13^ As scholars have noted, ^14^ despite the intention to control the theatre business, Parisian stages experienced a high level of dynamism, competition, and emulation. Taking inspiration from the official repertoires, which could not be repeated outside the *théâtres privilégiés*, unofficial theatres sought new and appealing ways to attract audiences through experimental rearrangements of the most popular subjects and repertoires performed in the official sites. Artists and performers had the freedom to jump from one theatre to another as a way to diversify their professional experience and find better economical arrangements.

**Figure 1.**
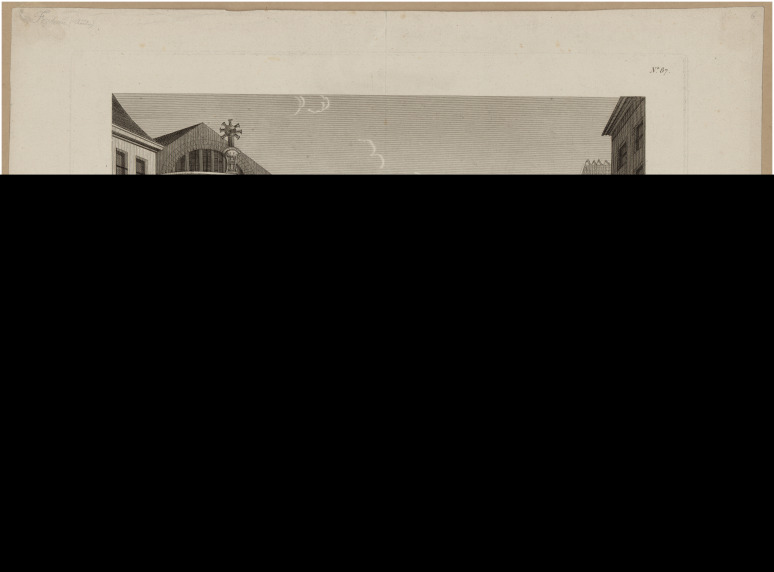
Eugène Dubois (graveur), Henri Courvoisier-Voisin (dessinateur), Basset Paris (editeur), *Rue Feydeau* ( ?), print, Musée Carnevalet, Histoire de Paris Musée Carnavalet, Histoire de Paris. Free from copyright.

An interesting case in this sense was that of the Théâtre de Monsieur (that in 1791 had its name changed to Théâtre Feydeau), an establishment opened in January 1789 sponsored by the Kings’ brother Louis-Stanislas Xavier who held the title of Monsieur. Despite its royal patronage, this theatre was not a *théâtre privilégié* holding a specific repertoire; instead, it was an experimental site of theatre production which employed striking visual effects that included spectacular stage settings, real animals on stage, and fireworks. Through the employment of a double cast of performers, an Italian troupe and a French one, its repertoire was inspired by the Neapolitan *opera buffa,* staged in its original language, but also re-arranged and re-casted for French audiences in the form of *pastiches* and *pieces à vaudeville*. ^15^ ( Figure 1)

The enactment of the Chapelier law in 1791 brought a halt to the system of privileges, allowing any current citizen with sufficient economic power to open a theatre, without repertoire limits. What followed was an exponential increase of theatre halls and in the city of Paris it was possible to count fifty of them, a situation that generated job offers and artistic mobility towards France, and particularly the capital. With the establishment of the Napoleonic Empire, theatre management and repertoires fell again under strict rules of state control, surveillance, and censorship. ^16^ A set of special decrees issued in 1807 reduced again the number of theatres to eight state-controlled establishments, with detailed regulations for their productions. ^17^

However, theatres were not the sole source of income for artists and performers. The latter could find jobs via an alternative circuit of popular entertainment. Among this it is worth mentioning the Revolutionary festivals organised in the 1790s ^18^ and the rich offer of *spectacles de curiosités*, which included animal exhibitions and performances like equestrian shows, magic lanterns, ventriloquism, puppeteering, and juggling as well as figure exhibitions. All these forms of entertainment remained highly popular during the Consulate and the Napoleonic Empire, allowing not only artistic exchange, but also the establishment of new forms of entertainment businesses like balls, cafés, concerts, and other shows. ^19^ In a way, instead of professionalism being tied to (and indeed) issued via court privilege, artists slowly became more aware that they could play an active part as “objects of trade and investment” in the society they were living in. ^20^

The section that follows highlights the vicissitudes of Italian artists that migrated from Italy to France in different moments of the eighteenth century: the Ruggieri brothers and Antonio Franconi moved to France during the Ancien Régime, the former in the late 1730s and the latter in 1756. Meanwhile, Ignazio Degotti moved to Paris in 1790, in the midst of the French Revolution. Hereby, my paper clarifies the reasons that made these artists leave their home country and the motivations that convinced them to stay in Paris, eventually transforming their art into a fruitful and long-lasting business.

### The Ruggieris: firework makers in search of job opportunities

Archival information on the early life of Gaetano (? – 1782), Pietro (? – 1778), Francesco (? – 1770), Antonio (? – 1776), and Petronio (? - 1794, most certainly the youngest) Ruggieri in Bologna is rare and fragmentary. This poses the problematic question of deciphering plans when they left Bologna and whether their initial aim was to permanently depart from their home country. Sources locate their arrival to France in the late 1730s ^21^, but their activity is better documented in the 1740s for their commitment to theatre, which took place at the Comédie-Italienne. This was one of the three *théâtres privilégiés* where Italian actors had established since 1660, performing a repertoire of French drama and Italian *commedia dell’arte*. Considering the prestige and the immediate success of their first theatrical employment, it is possible to argue that the Ruggieris had already established connections acting as “pull factors” to Paris, while an objective perspective of financial wealth was the “push factor” behind their decision to move.

At the Comédie-Italienne, the Ruggieri’s fireworks were employed not only as special effects within a theatre performance, but also as *per se* forms of spectacles that took the name of *spectacles pyrriques,* whose practical management was facilitated by an opening in the theatre ceiling that allowed smoke removal. ^22^ Ruggieri’s fireworks were performed accompanied by music and dance and repeated multiple times within a few months. Between 1743 and 1756, theatre historian Emanuele De Luca has traced over 29 fireworks spectacles performed with their own title, and therefore presented independently from a dramatic piece. Considering that they were performed within wood buildings, Ruggieri’s spectacles were perceived as reasonably safe and therefore, replicable. ^23^ Science historian Simon Werrett reports how audiences were fascinated by the fact that the Ruggieris pyrotechnical tricks took place without the presence of the technician on stage. Audiences could admire a fascinating variety of forms (girandoles, wheels, cascades polygons) sparkling in the air, without seeing any trace of human intervention, something that until then the French artificers could not do. ^24^

Their theatrical success drew King Louis XV’s attention, who in 1743 appointed them with the title of *Artificiers du Roi* and of the city of Paris. In 1749, Pietro, one of the eldest, was the first to become a naturalised French citizen for his remarkable service in the city of Paris, while Gaetano moved to London to work at the service of King George II. He settled in England, where in 1749 he became famous for organizing a marvellous spectacle in Saint James Park, for which he had recreated in the form of fireworks the burning of a palace originally designed by Servandoni while the orchestra played the suite *Music for the Royal Fireworks* composed by George Frederick Händel. ^25^

**Figure 2.**
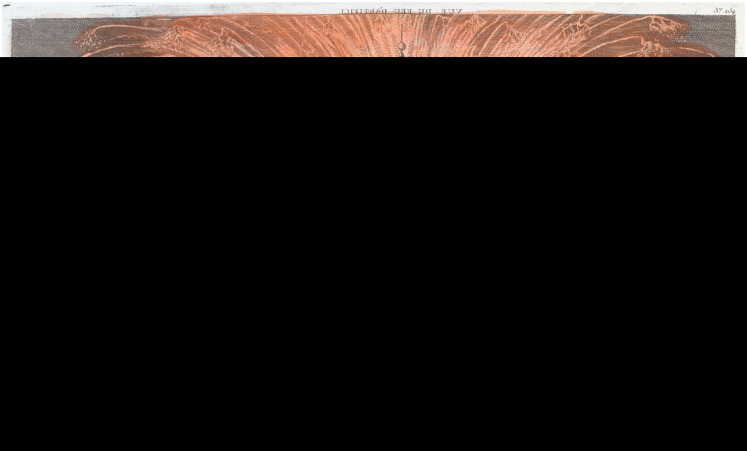
Author unkown (graveur), Basset Paris (editeur), *Feu d'artifice, tiré place Louis XV le 30 mai 1770* (1770), Musée Carnavalet Histoire de Paris. Free from copyright.

 The brothers that remained in France continued their career primarily working for the Royal family. Particularly famous for its fatal denouement, was the spectacle for the marriage celebrations of the Dauphin Louis Auguste (future Louis XVI) with the Archduchess Marie-Antoniette. For this feast, which took place on May 30 ^th^ 1770, the fireworks would have enriched a temporary setting consisting of a reproduction of the Temple of Hymen, the god of marriage. Preceded by a magnificent colonnade, the temporary architecture was installed near the statue of Louis XV, symbolizing the patronage and the blessing of the king upon the newly married couple. Due to miscalculations in safety management (errors, however, not attributable exclusively to the Ruggieris) one of the rockets fell upon the place where the reserve of explosives was stored. The explosion was intense and short-lived. At first glance the spectators believed they were witness to a new pyrotechnical trick. When the spectators realized they were in danger, they tried to escape but the crowd fell in a ditch leaving many people dead and wounded. ^26^ ( Figure 2)

Despite the fatal accident occurring during a major court celebration, the Ruggieris remained at the highest consideration of the Royal family who in April 1771, named Antonio and Petronio official *artificiers* du Comte de Provence, the kings’ brother who would sponsor the opening of the Théâtre de Monsieur years later. ^27^

**Figure 3.**
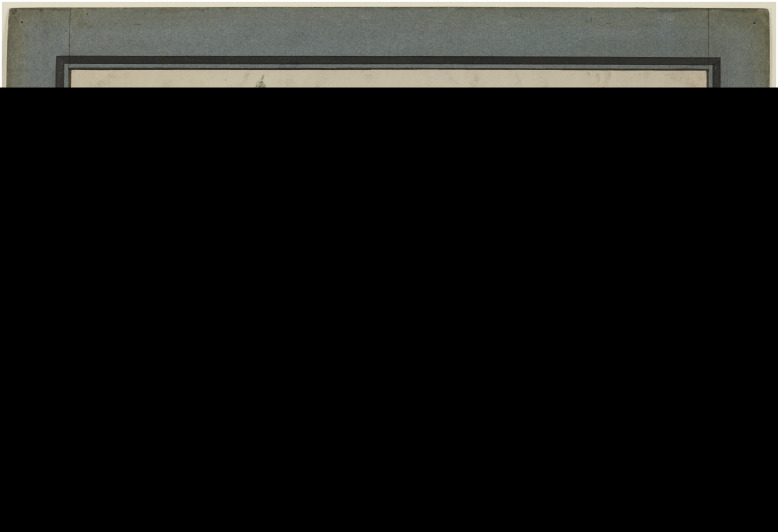
Jean-Jacques Huvé (père), *Face de l'escalier au droit du Saut de Niagara* (1770–1790 ca.), Musée Carnavalet Histoire de Paris. Free from copyright.

Their prestige was also reflected by a relatively wealthy financial situation. Shortly after their arrival in 1745, they had built their laboratory on Rue de Rully, nearby Les Halles, but a fatal accident and subsequent police advice forced them to install their workshop in more peripheral areas. ^28^ Following this accident Pietro (the eldest after Gaetano) speculatively invested in real estate in an area named *aux Porcherons,* near the current rue Saint-Lazare *.* He managed to acquire a building with large gardens, where in 1766 the brothers created a magnificent site of pleasure and entertainment which also included water attractions like a small replica of the Niagara’s falls:

Cet établissement consistait, sans parler des bâtiments, en un très beau jardin artistement disposé, Les spectacles, les jeux et les amusements qu’on y avait réunis formaient un ensemble agréable de fêtes auquel on a donné le nom de *fêtes champêtres*. Un très beau feu d’artifice terminait les plaisirs de la soirée. ^29^ ( Figure 3)

This investment, however, led them to circumstances that wavered between hype and financial discomfort, since the bills of a good part of their noble clients remained unsorted. During the 1770s, Petronio, the youngest among the brothers, started to take more control of the family affairs, remaining the only surviving sibling in France after Pietro’s death in 1778.

One of the most problematic aspects in the Ruggieri’s finances was that despite the diversification of the business, the greatest part of their revenue still relied on aristocratic commissions, which did not guarantee regularity of payments. Furthermore, the kingdom of Louis XVI, which started in 1774, was marked by a great financial crisis that caused cuts in the request of fireworks for state ceremonies. ^30^ Despite the king’s patronage remaining a major benefit, the diversification of demand beyond the circle of nobles and aristocrats, was a way to secure a more consistent source of income. In the Ruggieri's garden, firework displays were held on Thursdays, Sundays, and during festivities ^31^ and their entertainment offer was expanded to attract a larger audience who were keen on paying an entrance fee. This was something conceived as new, different, but also linked to scientific progress, like the launch of the first air balloon which took place in 1782 as a result of scientific experiments with hydrogen. The interest in science and chemistry had always been a crucial issue for the Ruggieris ^32^ and this aspect would remain important also when the second generation, carried on by Petronio’s two sons Claude Fortuné and Michel, took over the business.

Even though the Ruggieris’ success had been strictly linked to the royal family, for which they had created magnificent ephemeral spectacles accompanying weddings, funerals, and major court celebrations, the Revolution did not disrupt their business. Fireworks were never formally abolished in spite of their association with court feasts, since they were also used for moments of popular congregations, which became crucial during the Revolution. For example, Ruggieris’ fireworks were requested to celebrate the first anniversary of the fall of the Bastille. ^33^

Before the years of the Terror (1793–1794) that paralyzed Paris, the Ruggeris were also able to maintain commissions with the Théâtre de Monsieur. Here, they worked for some major productions like *Lodoïska* by Luigi Cherubini, which premiered successfully in July 1791 with stage settings made by the Italian designer Ignazio Degotti. For this opera, the Ruggeris created stunning special effects, consisting of a fire that had to destroy the whole scenery at the end of act III. The performance and its final conflagration were repeated several times within a month, each time leaving the audience astonished. ^34^

Then Petronio, the last member of the founding brothers, died in 1794 leaving his legacy to his two French-born sons, initially directed by a legal guardian named Monsieur Ducy. The generational switch coincided with the new social and political order that followed the Convention in 1792 and the death sentence of the monarchs in 1793. Ironically enough, most of the competitors in their field had disappeared after the Revolution, leaving only Claude Fortuné and Michel, the leading representatives of their art during the Consulate and the Napoleonic Empire.

Claude became famous for publishing the treaty *Élements de pyrotechnie, divisés en cinq parties,* first edited in 1802 and then re-published in 1811 and 1821. In this text Claude provides a recollection of the family experience, stressing in the preface how his job was not that of a simple technician or artisan, but comparing his experience to that of scientist, with competencies in physics, mechanics, and chemistry. ^35^ As previously outlined, the family had always shown great interest towards scientific progress, finding ways to associate it with their temporary and ephemeral forms of spectacle. None of the first generation, however, ever attempted to achieve a scientific aura through writing. Claude Fortuné dared to take it a step further, probably inspired by the great success of the *Traité élementaire de chimie* of Antoine-Laurent de Lavoisier, published in 1789. ^36^

The Napoleonic Empire and a new court protocol re-established a ritual of lavish feasts, most of them set up to celebrate military achievements. The Ruggieri's activity continued successfully until the end of the nineteenth century, with a family name that was turned into a brand associated with fireworks. Despite a change in the ownership the company still exists today as a leading enterprise in pyrotechnical entertainment. ^37^

From this analysis it is possible to argue that the story of the Ruggieri’s brothers is a story of migration embarked for objective reasons: proper connections in the court society of a new country (that we may identify as the “pull factor”) leading them towards the Comédie Italienne and the royal family with a clear possibility of financial wealth (“push factor”), which ultimately convinced them to stay. However, this case also has a strong subjective component: the Ruggieris left Bologna as a family of five siblings working in the same highly specialized field, and not as one single person chasing luck in a new country. From the beginning, the Ruggieris proposed themselves to the French market as an already organized family firm equipped to take part in the business and sociability of Paris, acting as agents of trade and investment in the new adopted country. ^38^ Moreover, like Pietro and Petronio did, when one of the family members experienced financial difficulties, another could intervene to support and to resume the family affairs. Their migration was of complete rupture with the home country, since a return to Italy was never contemplated by any of the five brothers.

### Antonio Franconi: a horse rider in need of movement

While there are some differences with the Ruggieris, the story of Antonio Franconi is also a matter of family business. Antonio (1737–1836), the founder, was born to a wealthy family in the Italian city of Udine. Around 1756 he killed his opponent during a duel and was forced to flee the country. Therefore, his decision to leave was a subjective move, dictated by the strong “push factor” consisting of the urgency of personal survival. He settled in France accompanied by his Italian wife Elisabetta, although there is no archival evidence of the “pull factors” that made him choose France instead of a different country. He led an itinerant life working as a horse rider and animal trainer, setting up his first shows with simple tricks made for a popular audience. Until 1783, he spent most of his time in Lyon, where he gained some success performing with trained pigeons.

In the same year, he attempted for the first time to find his way to Paris. ^39^ In the capital, however, the supply of performative entertainment was larger than in the provinces, with the result that it was difficult for Franconi to find a suitable spot for his business. In Paris though, he had the chance to meet Philip Astely, a horse rider from London and owner of the stable *l’Amphitéâtre Anglais*, sited in rue du Faubourg du Temple, where equestrian shows were assembled with juggling performances and acrobatic gymnastics. For Franconi, the meeting with Astely was a sort of training opportunity that gave him the understanding of the extent of possibilities that his business could take. Back in Lyon, he created his first stable, with a team that in 1790 consisted of 27 people, including his two French-born sons Henry (1779 – 1849), Laurent (1776 – 1849) (who both married two talented horse-riders) and 23 horses, transforming his activity into a small family enterprise under the name Franconis. ^40^

Until the Revolution, the Franconi's main business were the performances in Lyon and by frequent *tournées* in the surroundings. Tours were a challenging business of practical logistics and negotiation with the local authorities. Despite paying an exhibition fee, in each location the Franconis had to set a precise starting time. They had to make sure that their shows did not interfere with the timing of the performances scheduled in the local theatres. Nevertheless, on days when the theatre was closed - aided by a decision taken by the local authorities - they were afforded much more timing flexibility. ^41^

An interesting point emerging from the archival documentation is that Antonio Franconi was determined to propose his shows as something more than pure entertainment. In his views, his art could be an educational moment for everyone passionate about horsemanship. In 1787, during a tour in Nantes, the Franconis’ show was hosted as a great alternative to other types of entertainment that, according to the local administrator, were dangerous for corrupting the morals of the inhabitants, while also emptying their pockets. ^42^ At this stage, it can be argued that the blossoming of the family and their involvement in the business, together with the investment in over twenty horses and external staff, as well as the loyalty of the public during tours, were all factors that stabilized Antonio Franconi’s position in France, making his migration a definitive choice.

In 1793, in full revolutionary terror, a fire lit during the siege of Lyon destroyed the entire stable, forcing Franconi senior to ask for compensation from the Convention Nationale, the Revolutionary government established in 1792. On one hand, damages were officially recognized, and he was entitled to a reimbursement, but on the other, the Convention could not do anything to speed the slow process of issuing the money and therefore Franconi did not receive anything immediately. As a matter of coincidence, in the same year, due to the climate of uncertainty that followed the Revolution, Asteley decided to leave Paris and asked Franconi if he was interested in taking over his business. Supported by his family, on March 21 ^st^ 1793, the Franconis re-opened Astley’s Parisian stable under the new name of *Manège Franconi*. ^43^

In the capital, they also embarked on collaborations with the Théâtre Feydeau (former Monsieur), allowing some of their trained animals to show up on stage as special effects accompanying certain performances. This was the case, for example, of a small donkey that had to climb a high stage set representing a mountain, during *Eliza ou Le voyage aux glaciers du Mont Saint Bernard*, with music by Luigi Cherubini, text by Antoine de Révéroni de Saint-Cyr, and sets by the Italian stage designer Ignazio Degotti, which premiered in December 1794. ^44^

 After the establishment of the Napoleonic Empire, the country saw more theatrical and performative collaborations. The Franconis' equestrian skills were seen as a suitable match for events paying tribute to the power of the Great Army. Indeed, the use of trained horses in rich and lavish performances created at the Théâtre de l’Opéra (with stage sets by Degotti) to celebrate military campaigns was remarkable. For example, Franconis’ horses and riders were integrated in *Le Triomphe de Trajan,* first performed on 18 October 1807 with music by Jean-François Lesueur and Louis-Luc Loiseau de Persius and a libretto by Joseph-Alphonse Esménard, in time to celebrate the anniversary of Napoleon’s victory at the Battle of Jena-Auerstedt (14 October 1806). A similar situation was the engagement of 16 horses (plus riders) for the staging of *Fernand Cortez* on 28 November 1809, with music by Gaspare Spontini and libretto by Victor-Joseph-Étienne de Jouy and Joseph-Alphonse d'Esménard. The performance was conceived as a work of propaganda during the Spanish Campaign and the horses had to recall the battling of the Great Army, led by Napoleon, depicted as a contemporary conquistador emulating Hernán Cortez. ^45^

At the turn of the nineteenth century, the career of the Franconis was well established. In 1807 their stable was renamed *Cirque Olympique* and re-located in a site between rue du Mont-Thabor and rue Saint-Honoré. The troupe was expanded and new investments were made. In the summer, the family continued to perform on tour across the country, as during this season other forms of entertainment, *fêtes et bals champêtres,* distracted a large part of the Parisian public. ^46^ It is under the new denomination of *Cirque Olympique* that audiences started to define ‘circus’ as the type of entertainment offered by the Franconis: complex exhibitions performed with trained animals, jugglers, and acrobats.

Their locations became meeting points of artists, performers, and curious travellers, ^47^ while in the surroundings of their establishments, neighbouring shops and commercial activities benefited from the coming and going of people interested in the Franconis’ circus. In 1811, when the family attempted to relocate, moving to the surroundings of the Théâtre de la Porte Saint-Martin, over 50 shopkeepers signed a petition addressed to the Minister of the Interior, asking for intervention in finding a compromise between the interests of the Franconis and those of the retailers located in the neighbourhood. The controversial issue was solved under the agreement that Franconi senior would keep the *Cirque Olympique* in its location, while his children Henry and Laurent would move some of the activities to the new site of La Porte Saint-Martin. ^48^ In the same years (1810–1813), the family opened a third, temporary, establishment in the gardens of the Petit-Trianon.

**Figure 4.**
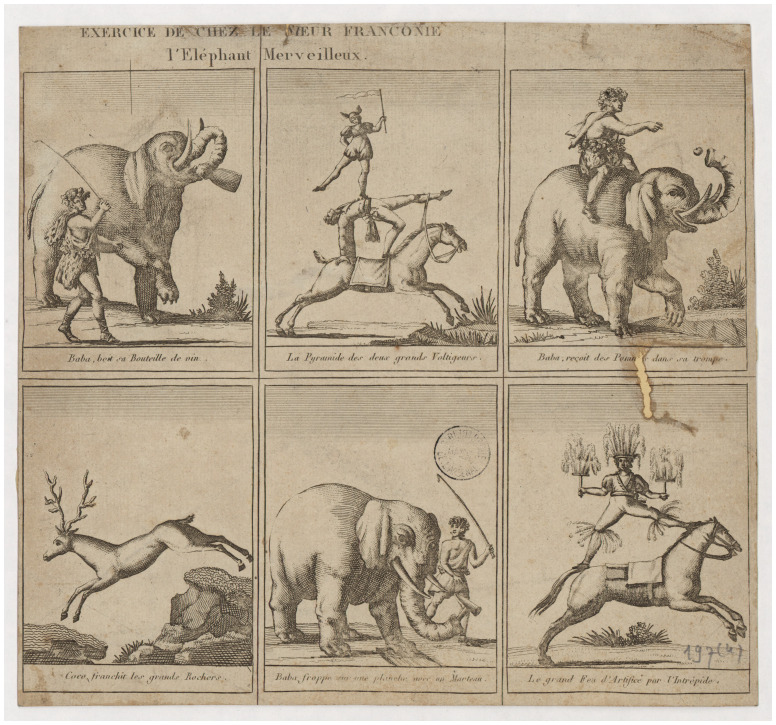
Author unknown, *Recueil sur exercices des Franconi au Cirque Olympique* (1807–1862), Paris, Bibliothèque Nationale de France. Free from copyright for non-commercial purposes.

This high level of engagement was made possible by a well-organized system of tasks and skills diversification between Franconi’s sons, who had involved their wives and trained their kids to join the business. Henri (whose nickname was Minette) was a brilliant mime and a choreographer of pantomimes, while Laurent was a vigorous horse-rider and acrobat, famous for creating equestrian shows that required great strength and flexibility. Among these, the most well-known was probably *Les Forces d’Hercules,* a number of Venetian origins ^49^ that he performed carrying three acrobats on his shoulders, while alternating trotting and galloping. Another one, performed in the 1820s, was the show called *arlequinades à grand transformation*, where Harlequin, the bouffon, pretended to use magic to transform objects or scenarios. The Franconis advertised this trick as something already popular in Astley’s circus in London, but less known in France. ^50^ Laurent had also inherited from his father a great talent in training animals, a talent that he applied not only to horses, but also with exotic animals, like the two elephants Baba and Kiounly and the deer Coco. ^51^ ( Figure 4)

Antonio died in 1836, almost reaching 100 years of age. Due to an illness, he lived the last two decades without sight, but he did not stop regularly attending the shows and being passionate about the innovations introduced by his sons. ^52^ Henry and Laurent both died in 1849, passing their artistic legacy to their children and grandchildren who successfully continued the Franconi’s circus until the early twentieth century.

It can be argued that Antonio Franconi migrated for subjective reasons, since his “push factor” was that he was forced to escape from his home country. The “pull factors” that moved him towards France remain unknown, but in his story, there are certainly many reasons that convinced him to stay in France and to experience a migration of complete rupture with his home country. First, despite starting his career performing mostly in Lyon and in the French provinces, his initial tours were relatively successful and sparked early loyalty within the public. Second, the involvement of his children and of his French extended family in his business strengthened his settlement in the adopted country. And third, he was always motivated by something more valuable than money: the idea that through his talent in horsemanship he could educate the public and create something innovative. In this respect, the first meeting with Astley and the following opportunity of a new start in Paris was certainly a major reason to stay until his passing, with a highly successful business that contributed to make circus performances known worldwide.

### Ignazio Degotti: a migration for proper connections

Ignazio Degotti (1758–1824) was born in Turin. His mother died giving birth to her second son when Ignazio was a year old; his father re-married shortly after and they had another boy, Ilario Antonio Degotti (1767 *–*1855) with whom Ignazio had a controversial relationship during adulthood.

Ignazio completed his apprenticeship with the Galliaris, a family of stage designers whose fame was acknowledged across Europe. In the 1780s Ignazio took working engagements in Rome and Naples, where his talent was appreciated, but where he never had the right chance to attain fame. In 1789, while in Naples, he received from Paris a letter by Giovan Battista Viotti (1755–1824), director and co-owner of the Théâtre de Monsieur, whom Degotti had met in Turin during childhood. Viotti invited him to Paris to create the stage sets of the Monsieur, an offer that certainly was a major “push factor”. The vision of personal success and the idea of working with Viotti, who already had fame and social prestige in a major capital of spectacle, were the “pull factor” that convinced the stage designer to move to Paris, despite receiving job offers from Madrid and London. ^53^

 As outlined in the introduction, since its opening, the Théâtre de Monsieur was a key, experimental site in the theatrical life of Paris and the chance of moving there certainly sounded appealing to Degotti. His career at the Monsieur was successful even after Viotti’s departure in 1792. Here, he created stage sets for three major productions with music by the resident composer Luigi Cherubini, such as *Lodoïska* (1791), *Eliza ou Le voyage aux glaciers du Mont Saint Bernard* (1794), and *Médée* (1797), working both with the Ruggieris (for *Lodoïska*) and with the Franconis (in *Eliza*). Even though he remained affiliated with the Monsieur, Degotti was able to experience artistic mobility towards other establishments, including a working trip to London's King’s theatre in 1797. In 1795 he took an engagement with the actor Talma and dramatist Jean-François Ducis for the staging of *Abufar ou La famille arabe* at the Théâtre de la République, for which he created magnificent and exotic settings inspired by the Arabian desert. ^54^ In the same year, he was appointed decorator in chief at the Théâtre de l’Opéra, reaching the fame and recognition he had coveted while in Italy. He maintained this position until the end of his career in 1822, despite some career breaks due to many quarrels with the administration, who blamed the painter for not poor time and budget management. ^55^

During the establishment of the Napoleonic Empire, Degotti also had the chance to work outside the theatrical engagements, entering the acknowledged circle of the painter Jacques-Louis David. Degotti collaborated with David at the design of the scenographic organisation of the large painting *The Coronation of Napoleon* ( *Le Sacre de Napoleon*, 1805–1807, Paris, Louvre), where his portrait stands right next to that of David, sealing a moment of major social prestige in Degotti’s career. ^56^ He was also the principal designer of the key and lavish imperial performances *Le Triomphe de Trajan* (1807) and *Fernand Cortez* (1808) *,* which had also involved Franconis’ horses. Despite the many quarrels he had with the Opéra administration for budget and time issues, Degotti’s stage sets were highly successful and appreciated by the audience.

Although his career in Paris started successfully, Degotti was in constant financial difficulties. In 1810, he temporarily resigned from his job at the Opéra, due to disputes with the theatre management. His colleague Jean-Baptiste Isabey (1767–1855), who was named in his place, wrote an interesting and generous letter to the director of the Opéra, proposing to cover the missing time owed to Degotti so that the pension might be afforded to him. Isabey was ashamed and mortified that Degotti was suffering, as the former was aware he had invested a great part of his income on books and art materials for his education over many years:

Monsieur Degoty [Degotti] n’a pu se faire sa fortune. Monsieur Degoty [Degotti] dépensait tout pour son instruction. Je sais de bonne part qu’il est on ne peut plus gêné! Je demande de nouveau de faire pour lui et comme si c’était lui les années qui lui restent à faire pour jouir de la pension à laquelle il aura droit de prétendre (…). ^57^

The reason for his financial misfortune was His younger brother Ilario, who worked as an assistant to Ignazio until the 1790s, was the reason for his financial misfortune. At the turn of the nineteenth century, Ilario returned to his hometown and embarked on financial speculations, involving Ignazio as guarantee. The investments failed, leaving Ignazio with many debts, and preventing him from returning home despite the difficulties he was experiencing in Paris.

 It is unclear how Degotti lived from 1810 to 1815. This moment, where he was officially off duty from the Opéra may have been a good chance to leave Paris, but the only information available for this time frame is that he had a sentimental relationship, but never married, nor had children. His relationship was a personal motivation that led him to continue his life in Paris, especially since his partner suffered from a severe illness and records state that he took great care of her and somehow, he was able to support her even financially. ^58^

In 1815, Degotti returned to the service of the Opéra, this time under a new management established by the restored monarchy. However, problems and misunderstandings with the administration persisted. Through a reinvigorated system of court connections, Viotti also returned to Paris in 1819 as director of the Opéra, in an engagement that lasted until 1821. ^59^ In 1818, Degotti wrote a letter to Viotti, recalling the fact that more than thirty years earlier the musician had contacted the painter while he was in Naples and had insisted on hiring him for the Monsieur. Degotti wanted Viotti, whom he calls a “compatriot”, to return the favour by giving him some protection and better working conditions at the Opéra. This situation never happened and in 1822 Degotti resigned again from his position, this time for the last time.

The case of Degotti, is a completely different migration story from that of the Ruggieris and of the Franconis. Degotti had a clear subjective reason to choose Paris (instead of London, and Madrid for example); in his case the “push” and “pull” factors seem to almost coincide in Viotti’s offer. For Degotti, the invitation itself carried social prestige given Viotti’s privileged position as director and co-owner of a famous theatre establishment. Degotti must have seen in his compatriot a “push factor” and a fast path to success and towards a rewarding job position, which certainly were also major “pull factors” behind his move. His intuition was correct since his career started successfully, inducing him to remain in Paris. Even when he was appointed at the Opéra and circumstances changed and became more problematic, he decided to stay. He did not consider returning home to solve his financial problems because he was confident that sooner or later things in Paris would return to be positive as they were when he started his career. He maintained this hope until 1819, when he wrote to Viotti for support. The crucial element that makes his story different from that of the Ruggieris and of the Franconis, is that Degotti never had a family, or children who were able to support him both financially and in artistically, since his brother, who worked with him until the 1790s was a problem, rather than an asset.

## Conclusions

Migration stories of artists that worked with ephemeral artistic expressions have often remained a neglected aspect in the history of theatre and spectacle. The three cases here occurred at different moments of the eighteenth century. In all three stories the protagonists experienced a migration of complete rupture with their home country and were influenced by different “push” and “pull factors” as in Markovits definition.

For the Ruggieris, who moved during the Ancien Régime seeking better financial and working engagements, the patronage of the royal family was the key element to success. However, this was not sufficient to financially maintain the business. A good dose of entrepreneurial skill was crucial during difficulties and allowed them to turn their family name into a powerful brand associated with fireworks. Their settlement in France was facilitated by the fact that they were an already organised family enterprise able to sustain each other in case of financial need. The brothers married in Paris and welcomed additions to the family. The second generation progressed the business until the late nineteenth century, but this was already a French story, rather than Italian.

To some extent, their story has some similarities with that of the Franconis, even though Antonio, the founder of the family, never benefited from the same royal sponsorship granted to the Ruggieris. For Franconi, the reason behind his move was the need to escape from his home country. In France he slowly began to settle his business, creating loyalty among the public. The turning points of his career were the partnership with Astely and the degree of interest that the Napoleonic spectacle had towards equestrian shows. However, what made Antonio stands out was the ambition and the determination to create shows beyond pure entertainment, moments of spectacle that could be new, but also educational. This mission was transferred to his French-born children, who successfully managed the affairs until the end of the nineteenth century. In the wake of this analysis, it is possible to state that both for the Ruggieris and for the Franconi, the key element for a successful business was the family management of their artistic skills and investments.

Instead, Degotti’s story is different. The reason behind his move was the invitation by Viotti, a clear “push factor” that attracted him to France, where he wanted to improve his career and find social prestige (“pull-factors). In Paris, he was encouraged by a well-received initial success and by an established network of important connections, like with the painter David. These circumstances were much more - and certainly, sufficiently - powerful to motivate Degotti to persist in living and working on in Paris, despite the many arguments with the Opéra administration. For him, the family element was a problem rather than an advantage since his partner died without leaving him children and the financial problems created by his brother Ilario prevented him from going back to Italy and forced him to live in economic hardship. Despite that, he continued to live in Paris, hoping his situation would improve.

Migration of artists of course continues to this day, and it would be an interesting point of departure for research in comparative histories of migration. Have push and pull factors changed over time? who are the neglected or subordinated artists of our contemporary times?

## Ethics and consent

Ethical approval and consent were not required.

## Data Availability

The data for this article consists of bibliographic and archival references, which are included in the footnotes.

## Footnotes

^1^ Mélanie Traversier (ed.), “Musiques nomades : objets, réseaux, itinéraires. Europe, XVII ^e^-XIX ^e^ siècle” *Diaspora* 26 (2016) Publisher Full Text ; in this volume see especially David Do Paço, “Mobilités et précarités : Lorenzo Da Ponte dans le monde de l’opéra (1779-1830)”, pp. 115–132. See also Tatiana Korneeva (ed.), *Mapping Artistic Networks: Eighteenth-Century Italian Theatre and Opera Across Europe* (Turnhout: Brepols, 2022) Reference Source

^2^ Martine de Rougemont, *La vie théâtrale en France au XVIIIème siècle* (Paris: Honoré Champion, 1988) Reference Source ; Robert M. Isherwood “Entertainment in the Parisian Fairs in the Eighteenth Century”, *The Journal of Modern History 53,* 1 (1981): 24–48 Publisher Full Text

^3^ Pierre-Yves Beaurepaire, Philippe Bourdin and Carlotta Wolff (eds), *Moving scenes. The circulation of music and theatre in Europe 1700–1815* (Oxford: Voltaire Foundation, 2018) Reference Source ; Reinhart Strohm (ed.), *The Eighteenth-Century Diaspora of Italian Music and Musicians* (Turnhout: Brepols, 2001) Reference Source About mobility to Paris: Barbara Nestola, “Italian Music, French Singers. Reception and performance practice on the Parisian stage at the Beginning of the 18th century”, in Alessandro Di Profio and Damien Colas (eds.), *D’une scène à l’autre. L’opéra italien en Europe,* vol. 1 *, Les pérégrinations d’un genre* (Liège: Mardaga, 2009), pp. 253-266 Reference Source On the topic of dance see Arianna Fabbricatore, ‘La confrontation italo-française sur la danse théâtrale au XVIIIe siècle : rivalités, contaminations et jeux de pouvoir’, *Revue des Études Italiennes* 59 (2013) : 77–88 Reference Source

^4^ A recent attempt in the study of people engaged with ephemeral forms of spectacle is the publication by Dominique Bauer and Camilla Murgia (eds.), *Ephemeral Spectacles, Exhibition Spaces, and Museums 1750–1918* (Amsterdam: Amsterdam University Press, 2021) Reference Source

^5^ Daniel Roche, Humeurs vagabondes, chapter 13 (Paris : Fayard, 2003), pp. 859–921 Reference Source

^6^ Mark Darlow, *Staging the French Revolution: Cultural Politics and the Paris Opera 1789–1794,* (Oxford; New York, Oxford University Press: 2012), p. 5 Reference Source

^7^ Rahul Markovits, *Staging Civilization,* trans. By Jane Marie Todd (Charlottesville; London, University of Virginia Press: 2021) Reference Source

^8^ *Ibid*., p. 32, 38–39

^9^ *Ibid.*, p. 38–39

^10^ Jean-François Dubost, *La France Italienne XV ^e^ - XVII ^e^* siècle. *Préface de Daniel Roche* (Paris: Aubier, 1997) Reference Source

^11^ Marie-Noëlle Matuszeck, «La France Italienne», *Bibliothèque de l’école de chartes* 157/1 (1999): 273–276 Publisher Full Text

^12^ Jérôme de la Gorce, *Carlo Vigarani intendant des plaisirs de Louis XIV* (Versailles: Editions Perrin et Château de Versailles, 2005) Reference Source; Michèle Sajous D’Oria, *Servandoni maître des machines* (Paris: Classiques Garnier, 2022) Reference Source . See also Mathias Auclair ‘Architects italiens et peintres français. Les décorateurs de l’Opéra de Paris sous l’Ancien Régime’ in Mickael Bouffard, Christian Schirm, and Jean-Michel Vinciguerra (eds.) *Un air d’Italie. L’Opéra de Paris de Louis XIV à la Révolution* (Paris: Bibliothèque nationale de France, 2019), pp. 148–164 Reference Source

^13^ Mark Darlow ‘L’effritement du privilège théâtral les débats de 1789–1790’, Sabine Chaouche, Denis Herlin, and Solveig Serre (eds.) *L’Opéra de Paris, la Comédie-Française et l’Opéra-Comique: Approches comparées (1669–2010)* (Paris: Publications de l’École nationale des chartes, 2012) pp. 61–73 Publisher Full Text

^14^ Emanuele De Luca, and Barbara Nestola, ‘Parcours transversaux pour une relecture du spectacle parisien sous l’ancien régime’, *Revue d’Histoire du Théâtre* 289 no 1 (2021): 6–14 Publisher Full Text

^15^ Alessandro Di Profio, *La révolution des Bouffons* (Paris: CNRS éditions, 2003) Reference Source ; Andrea Fabiano, Michel Noiray, *L’opera italiana in Francia nel Settecento* (Torino: EDT, 2013) Reference Source

^16^ David Chaillou, « À la gloire de l'Empereur : l'opéra de Paris sous Napoléon I ^er^ », *Napoleonica. La Revue*, 7 (2010/1): 88–105. DOI : 10.3917/napo.101.0088 Publisher Full Text

^17^ Jacques Antoine Dulaure, *Histoire physique, civile et morale de Paris (…),* 2 ^nd^ ed., vol. 9 (Paris: Guillame, 1824), p. 266–267 Publisher Full Text

^18^ Mona Ozouf, «Symboles et fonction des âges dans les fêtes de l’époque révolutionnaire», *Annales historiques de la Révolution française* 202 (1970): 569–593 Publisher Full Text

^19^ Nicole Wild, «Les spectacles de curiosité du Premier au Second Empire», *Vibrations*, 5 (1988): 3–28 Publisher Full Text; Cyril Triolaire, «Fêtes officielles, théâtres et spectacles de curiosités pendant le consulat et l’Empire dans le 11ème arrondissement théâtral impérial – pouvoir, artistes et mise en scène», *Annales historiques de la Révolution française* 362 (2010): 131–142 Publisher Full Text. I also signal the research project managed by the Centre d’Histoire « Espaces et Cultures » (CHEC), Project website

^20^ This definition appears in Pierre-Yves Beaurepaire and Charlotta Wolff “Introduction”. In: Beaurepaire, Bourdin and Wolff, *Moving scenes,* pp. 1–6, here: p. 2 Reference Source

^21^ In this article names are used in Italian and not in their French version. Luigi Montanari, «La pirotecnia ed i fratelli Ruggieri da Bologna», *Strenna Storica Bolognese* 30 (1980): 235–24, p. 237 Reference Source

^22^ Simon Werrett, *Fireworks: Pyrotechnic Arts and Sciences in European History* (Chicago: University of Chicago Press, 2010), pp. 139–153; here 140 Reference Source

^23^ Emanuele De Luca, «Il repertorio della Comédie-Italienne di Parigi (1716–1762); Le répertoire de la Comédie-Italienne de Paris (1716–1762)» in *Les savoirs des acteurs italiens*, digital collection directed by Andrea Fabiano for the interdisciplinary programme *Histoire des Savoirs* Publisher Full Text, p. 19. De Luca is currently working on new research projects about the theatrical engagements of the Ruggieris at the Comédie-Italienne.

^24^ Werrett, *Fireworks*, p. 142 Reference Source

^25^ Armando Fabio Ivaldi, Memorabili *e sfortunati «Fuochi d’Allegrezza» a Green Park (1749): una festa reale con grandiosa «macchina» di Jean-Nicolas Servandoni, «Maestri Bombardieri» italiani e musica di Georg Friedrich Händel,* Iskrena Yordanova, Giuseppina Raggi (eds.). Cadernos de Queluz vol. 3 *Theatre Spaces for Music in 18th-Century Europe* (Wien: Hollitzer Verlag, 2020), pp. 445–482 Reference Source

^26^ Louis Petit de Bachaumont, Jules Amédée Désiré Ravanel, *Mémoires secrets de Bachaumont de 1762 à 1787,* vol. 3 (Paris: Imp. Fournier, 1830), pp. 157–159 Publisher Full Text

^27^ Paris, Archive Nationales, O/1/117, fol. 473 Reference Source

^28^ Paris, Archives Nationales, Y//9499, pièce n° 819 Reference Source

^29^ “Without mentioning the buildings, this establishment consisted of a very beautiful and artistically arranged garden. The performances, games, and amusements here gathered formed a pleasant set of feasts to which we gave the name of *fêtes champêtres*. A beautiful firework concluded the pleasures of the evening.” Lucien Lazard, *Deux Jardins Disparus* (Paris: Extrait du Bulletin de la Société Le Vieux Montmartre, 1895), pp. 3–4 Publisher Full Text Traslation are by the autor.

^30^ Patrick Bracco, Elisabeth Lebovici, *Ruggieri 250 ans de feux d’artifice* (Toulouse: Denoël, 1988), p. 49 Reference Source

^31^ Lazard, *Deux Jardins Disparus*, p.6 Publisher Full Text

^32^ Werret, *Fireworks*, pp. 220–231 Reference Source

^33^ Werret, *Fireworks*, p. 226 Reference Source

^34^ Nicholas Daly, “Fire on Stage”, 19 *Interdisciplinary Studies in the Long Nineteenth Century* 25 (2017) Publisher Full Text Elisa Cazzato, “Ignazio Degotti Staging Luigi Cherubini: Making Visual Culture at the Théâtre Feydeau”, *Cherubini Studies,* vol. 3: *Il teatro musicale di Luigi Cherubini,* Maria Teresa Arfini, Francesca Menchelli-Buttini, Emilia Pantini (eds.) (Würzburg: Königshausen & Neuman, 2020), pp. 225– 242 Reference Source

^35^ I refer here to the third edition : Claude-Fortuné Ruggieri, *Élements de pyrotechnie, divisés en cinq parties, 3eme éd.* (Paris: Chez Barba, 1821) Pubisher Full Text

^36^ Antoine Laurent de Lavoisier, *Traité Elémentaire de Chimie*… (Paris: chez Cuchet, 1789) Publisher Full Text

^37^ Company Website

^38^ Beaurepaire, Bourdin, and Wolff (eds), *Moving scenes,* p. 2 Reference Source

^39^ Frédéric Hillemacher, *Cirque Franconi* (Lyon: Imp. Perrin & Marinet, 1875) Publisher Full Text

^40^ Paris, Archives Nationales, D-IV-37 Reference Source

^41^ *Ibidem.*

^42^ *Ibidem*.

^43^ The establishment change location several times. For further references see the accurate work of Caroline Hodak Project Website

^44^ Paul Smith (pseudonyme of Euduard Monnais), *Esquisses de la vie d'artiste* (Paris: 1844), p. 356–357 Publisher Full Text

^45^ Elisa Cazzato, “Performing for Napoleon: Production Quarrels at the Paris Opéra”, *Dance Research,* 41.2 (2023): 275–296 https://doi.org/10.3366/drs.2023.0407 Publisher Full Text

^46^ Paris, Archives Nationales, F-21-1142, cart. 1809–1823, *Paris le 3 Juin 1813 Rapport au ministère de l’Intérieur*, n° 403 Reference Source

^47^ Juan Plazaola, *Le baron Taylor: portrait d’un homme d’avenir* (Paris: Fondation Taylor, 1989), p. 41 Reference Source

^48^ Paris, Archives Nationales, F-21-1142, folder 1810–1817, *Brevet d’exportation* Reference Source

^49^ Giuseppe Tassini, Curiosità veneziane…, vol. II (Venezia: premiata tipografia Gio Cecchini, 1863), p. 153 Reference Source

^50^ Paris, Archives Nationales, F-21-1142, folder 1823–1826, *Lettre des Franconi frères à son excellence.* Reference Source

^51^ Hillemacher, *Cirque Franconi*, p. 14–15, 25 Publisher Full Text

^52^ *Ibidem*., p. 17–18 Publisher Full Text

^53^ Elisa Cazzato, “Chronicles of two Piedmontese in Paris: the stage designer Ignazio Degotti (1758–1824) and the composer Gian Battista Viotti (1755–1824)’. In: Iskrena Yordanova, Cristina Fernandes (eds.) Cadernos de Queluz vol. 4, “Padron mio colendissimo…”Letters about Music and the Stage in the 18th Century (Wien: : Hollitzer Verlag, 2021), pp. 655–704 Reference Source

^54^ Martial Poirson, “Scènes d’Orient, regards d’Occident: Images et imaginaire orientaliste dans Abufar ou La Famille arabe (1795) de Ducis” In: *Image et voyage: Représentations iconographiques du voyage, de la Méditerranée aux Indes orientales et occidentales, de la fin du Moyen Âge au XIXe siècle* (Aix-en-Provence: Presses universitaires de Provence, 2012), pp. 203–212 Pubisher Full Version

^55^ Mathias, Auclair « L'atelier des décors de l'Opéra (1803–1822) », *Revue de la BNF*, vol. 37, no. 1, 2011, pp. 5–10 Publisher Full Text

^56^ Mark Ledbury, “Musical mutualism, David, Degotti and operatic painting”. In: *Art, Theatre and Opera in Paris, 1750 – 1850. Exchanges and Tensions,* eds. Sarah Hibberd, Richard Wrigley, Farnham, Ashgate, 2014, pp. 53–76 Reference Source

^57^ “Mr. Degoty [Degotti] could not make his fortune. Mr. Degoty [Degotti] spent everything for his education. I know as a fact that he is very embarrassed! I ask again to do for him and as if it were the years that remain to him to enjoy the pension to which he will be entitled to claim.” Paris, Bibliothèque de l’Opéra, *Dossier Isabey*, *Lettre de Isabey, 21 aôut 1810* Reference Source

^58^ Elisa Cazzato, An Italian Artist in Paris: The career and designs of Ignazio Degotti (1758–1824), PhD Diss., The University of Sydney, 2017, pp.175–179 Reference Source

^59^ Warwick Lister, *Amico* (Oxford : Oxford University Press, 2009) pp. 317–47 Reference Source

