# Supplementary material for: The lure of the foreign stage: Chronicles of Italian mobility to France in the long eighteenth century
Source: Open Res Eur. 2024 Feb 22;4:38. [Version 1] doi: 10.12688/openreseurope.17027.1 (PMC10958156; doi:10.12688/openreseurope.17027.1)
Supplement: Peer review history

# Reviewer report: Reviewer response for version 1

**DOI:** 10.21956/openreseurope.18400.r38589

**Published:** 2024-03-21

**Authors:** Bruno Forment

**Affiliations:** 1 Orpheus Institut, Ghent, Belgium

This article explores the migration patterns of Italian spectacle to the cultural milieu of Paris in a transformative period, the late eighteenth and early nineteenth century. By offering a comparative and connective view on the lives and careers of three individuals engaged in as many different forms of entertainment (fireworks, circus, and stage design), the author escapes the pitfalls of narratives dealing with prominent figures and well-studied artistic traditions.

 Overall, the essay is well constructed and written, but some sections could benefit from further refinement. Four areas of research could be enhanced.

 Firstly, the integration of concepts (“push” and “pull” factors; “objective” and “subjective” motivations) from Rahul Markovits’ work on mobility in early modernity is rather light, suffering somewhat from being inserted into a concatenation of biographical anecdote. Thus, the explanation surrounding Antonio Franconi’s “decision to leave” as a “subjective move, dictated by the strong “push factor” consisting of the urgency of personal survival” lacks depth. The statement “[a]round 1756 he killed his opponent during a duel and was forced to flee the country” leaves the reader wanting for a more nuanced exploration of the contextual factors influencing Franconi’s decision. At that time, Udine (Friuli) was still part of the Venetian Republic; it could have sufficed for Franconi to migrate to nearby, Habsburg-ruled Milan (Lombardy) to escape imprisonment and continue his career in a more favorable theatrical context.

 Secondly, Paris could be distinguished more sharply from other migrational destinations for artists, most notably London (where Gaetano Ruggeri would become active, and where Ignazio Degotti operated for a brief time) and Amsterdam. For example, it could be argued that the combination of liberal impresarial activity and enduring aristocratic structures governing Paris’ socio-cultural networks (cf. the *théâtres priviligiés*) were more attractive to Italians (familiar with feudal-aristocratic patronage) than London and Amsterdam’s liberal-capitalistic structures, which affords a relatively lesser degree of professional security. It might be symptomatic, in this regard, that “the Ruggieris’ success had been strictly linked to the royal family,” as the author notes, while Paris simultaneously offered “Artists and performers … the freedom to jump from one theatre to another as a way to diversify their professional experience and find better economical arrangements.”

 Thirdly, while the author cites a number of previous studies on artistic migration (see, for example, notes 3 and 10), I would advise her to also consider Shearer West’s edited volume *Italian Culture in Northern Europe in the 18th Century* (Cambridge: Cambridge University Press, 1999), which considers the topic of Italian cultural diaspora from a broader, multidisciplinary angle, also covering religious (cf. Catholic Paris versus Protestant London), political, linguistic, and esthetic factors. The author of this article is certainly well aware of the long history of Italian migration to Paris, yet I was hoping to read more about the longevity of Italian ‘colonies’ in the French capital, which must have exerted a substantial “pull.”

 Fourthly, it is regrettable that this article relies mostly on secondary sources and incorporates relatively little new archival work. For instance, it is justly mentioned that “[a]rchival information on the early life of Gaetano (? – 1782), Pietro (? – 1778), Francesco (? – 1770), Antonio (? – 1776), and Petronio (? - 1794, most certainly the youngest) Ruggieri in Bologna is rare and fragmentary,” but readers are not told why this is so. Knowledge about familial networks and related matters can be enhanced substantially through research in parochial, notarial or other registers, as numerous precedents illustrate (e.g., Milada Jonášova, ‘I Denzio: tre generazioni di musicisti a Venezia e a Praga’, *Hudební věda* 45/1-2 (2008), 57-114; Micky White, *Antonio Vivaldi: A Life in Documents* (Florence: Olschki, 2013); Gianluca Stefani, ‘Nuovi documenti su Domenico Mauro e figli’, in *Illusione scenica e pratica teatrale. Atti del convegno internazionale di studi in onore di Elena Povoledo (Venezia, 16-17 novembre 2015)*, ed. Maria Ida Biggi (Firenze: Le Lettere, 2016), 284-293). If the author is unable to travel to Bologna for the necessary investigations, she can always rely on citizen science, getting into touch with local genealogists.

 Finally, we would advise to author to have the essay edited to improve certain unfortunate phrasings (e.g., “into scholarship account”; “Théâtre de Monsieur (that [which] in 1791 had its name changed)”; “Hereby [?], my paper clarifies the reasons …“; “The explosion was intense and short-lived [?]”; “For this opera, the Ruggeris created stunning special effects, consisting of a fire that had to destroy [really?] the whole scenery at the end of act III”; “Ilario Antonio Degotti (1767 *–*1855) with whom Ignazio had a controversial [troubled?] relationship during adulthood”; “Reinhart [Reinhar d] Strohm”).

 If a revision can take these remarks into account, I am positively sure this article can become an outstanding contribution to research on artistic migration in the long eighteenth century.

Is the argument persuasive and supported by appropriate evidence?

Yes

Is the work clearly and cogently presented?

Yes

Is the topic of the essay discussed accurately in the context of the current literature?

Partly

Does the essay contribute to the cultural, historical, social understanding of the field?

Partly

Reviewer Expertise:

music history, theater history, scenography

I confirm that I have read this submission and believe that I have an appropriate level of expertise to confirm that it is of an acceptable scientific standard, however I have significant reservations, as outlined above.

# Reviewer report: Reviewer response for version 1

**DOI:** 10.21956/openreseurope.18400.r38591

**Published:** 2024-03-21

**Authors:** Elena Mazzoleni

**Affiliations:** 1 Universita degli Studi di Bergamo, Bergamo, Lombardy, Italy

The article investigates the migration of three Italian-born artists working in France in the field of ephemeral entertainment, Franconi, Ruggeri brotrhers, Degotti. They took part to

 a broader process of cultural history and artistic mobility of the long eighteenth century. Due to the immaterial and contingent medium in which these artists chose to work, the mobility of these artists has often remained a neglected story.  The paper argues that their lives and their artistic expertise were not only aesthetically relevant, but also very much integrated within the defined social and cultural context.

 The article is clearly presented. It partially contributes to the cultural understanding of the field; i.e. on Franconi the bibliography is wide, especially on the migration of this circus. Therefore, the argument is not persuasive enough and not always supported by appropriate evidence. The author has not brought any quotations from the sources. The arguments result is not being supported enough, so the essay is not persuasive; it is rather descriptive.

 The bibliographical sources considered on the subject are really partial. I suggest to take into account the English, American and Italian bibliography, which are wide, especially on Franconi's career. The author just or mainly mentions French sources.

 On Franconi and Ruggeri, I also suggest to deepen the subjects and make profitable relationships for instance with the context of the Parisian and European circus of the time and Foire theatre.

Is the argument persuasive and supported by appropriate evidence?

Partly

Is the work clearly and cogently presented?

Yes

Is the topic of the essay discussed accurately in the context of the current literature?

Partly

Does the essay contribute to the cultural, historical, social understanding of the field?

Partly

Reviewer Expertise:

18th and 19th popular theatre, circus, vaudeville and pantomime

I confirm that I have read this submission and believe that I have an appropriate level of expertise to confirm that it is of an acceptable scientific standard, however I have significant reservations, as outlined above.

# Reviewer report: Reviewer response for version 1

**DOI:** 10.21956/openreseurope.18400.r38590

**Published:** 2024-03-21

**Authors:** Tatiana Korneeva

**Affiliations:** 1 Norwegian University of Science and Technology (NTNU), Trondheim, Norway

The European mobility of eighteenth-century Italian theatre professionals (musicians, playwrights, actors, set designer), which was decisive for cultural transfer and exchange, is a phenomenon of European scale. The European success of Italian theatre and opera can be attributed toe importance of authors such as Pietro Metastasio and Carlo Goldoni, to the widespread use of the Italian language as a means of communication within the European aristocracy, and to the itinerant careers of many Italian-born artists (especially, singers and composers).

 Certainly, the phenomenon of the mobility of Italian-born theatre professionals to France and other European countries is well known to music, theatre, and art historians: This is evidenced by the extensive bibliography cited in this essay. However, this article has the merit of providing a significant change of perspective by focusing on the mobility of those who worked as providers of ephemeral forms of entertainment such as fireworks, circus performances, and stage sets, which has remained a rather neglected story.

 By analysing visual and written archival sources, this article focuses on the migration histories of three Italian spectacle makers who moved from Italy to France at different moments of the eighteenth century: the fireworks technicians Ruggieri brothers, the circus performer Antonio Franconi and his family, and the stage designer Ignazio Degotti. This article sheds light on their stories of mobility and reveals the reasons why they emigrated and the motives that led them to stay in France. The author concludes by explaining how their lives and artistic expertise were embedded in the social and cultural context they choose to live in.

 This essay is an original, solid, informative, and well-researched scholarly work suitable for publication in Open Research Europe *. *The narrative flows naturally and smoothly. The overall structure of the essay is very clear and effective, and it can be published as is, without stylistic or content revisions.

 My only suggestion to the author is to uniform the spelling of “royal family”. Sometime “royal” is written with big caps (p. 6), sometimes with small. Also, the sentence on page 12 does not read well. “The reason for his financial misfortune was His younger brother Ilario, who worked as an assistant to Ignazio until the 1790s, was the reason for his financial misfortune”: “His” should be taken in small caps, and it seems that “and” after “1790s” is missing.

Is the argument persuasive and supported by appropriate evidence?

Yes

Is the work clearly and cogently presented?

Yes

Is the topic of the essay discussed accurately in the context of the current literature?

Yes

Does the essay contribute to the cultural, historical, social understanding of the field?

Yes

Reviewer Expertise:

Theater Studies, Comparative Literature

I confirm that I have read this submission and believe that I have an appropriate level of expertise to confirm that it is of an acceptable scientific standard.
